# Evaluation of the Quality of Guidelines for Myasthenia Gravis with the AGREE II Instrument

**DOI:** 10.1371/journal.pone.0111796

**Published:** 2014-11-17

**Authors:** Zhenchang Zhang, Jia Guo, Gang Su, Jiong Li, Hua Wu, Xiaodong Xie

**Affiliations:** 1 School of Basic Medical Sciences, Lanzhou University, Lanzhou, P. R. China; 2 Department of Neurology, The Second Hospital of Lanzhou University, Lanzhou, P. R. China; Jilin University, China

## Abstract

**Background:**

Clinical practice guidelines (CPGs) are systematically developed statements to assist practitioners in making decisions about appropriate healthcare in specific clinical circumstances. The methodological quality of CPGs for myasthenia gravis (MG) are unclear.

**Objective:**

To critically evaluate the methodological quality of CPGs for MG using AGREE II instrument.

**Method:**

A systematical search strategy on PubMed, EMBASE, DynaMed, the National Guideline Clearinghouse (NGC) and the Chinese Biomedical Literature database (CBM) was performed on September 20th 2013. All guidelines related to MG were evaluated with AGREE II. The software used for analysis was SPSS 17.0.

**Results:**

A total of 15 CPGs for MG met the inclusion criteria (12 CPGs in English, 3 CPGs in Chinese). The overall agreement among reviews was moderate or high (ICC >0.70). The mean scores (mean ± SD) for al six domains were presented as follows: scope and purpose (60.93% ±16.62%), stakeholder involvement (40.93% ±20.04%), rigor of development (37.22% ±30.46%), clarity of presentation (64.26% ±16.36%), applicability (28.19% ±20.56%) and editorial independence (27.78% ±28.28%). Compared with non-evidence-based CPGs, evidence-based CPGs had statistically significant higher quality scores for all AGREE II domains (P<0.05). All domain scores appear slightly higher for CPGs published after AGREE II instrument development and validation (P>0.05). The quality scores of CPGs developed by NGC/AAN were higher than the quality scores of CPGs developed by other organizations for all domains. The difference was statistically significant for all domains with the exception of clarity of presentation (P = 0.07).

**Conclusions:**

The qualities of CPGs on MG were generally acceptable with several flaws. The AGREE II instrument should be adopted by guideline developers, particularly in China.

## Instruction

Myasthenia gravis (MG) is caused by antibody-mediated autoimmunity against the nicotinic acetylcholine (ACh) receptor (AChR) at the neuromuscular junction (NMJ). The prevalence of MG in the United States is estimated to be approximately 20/100,000 population and it occurs in all genders, ethnicities and ages [1]. MG is characterized by varying degrees of weakness and rapid fatigue of skeletal and voluntary muscle groups. The ranging of the clinical course of MG is from remission in an early stage to acute exacerbation and even death [2]. In the past, diagnosis, treatment and prognosis have been remarkably improved due to the development of successful surgical and pharmacological treatments. However, there remains a notable challenge regarding spontaneous remission [3], [4]. Currently, several guidelines for MG are available. However, in common with many other fields and medical disciplines, only a minority of physicians fully comply with guidelines and recommendations are slow to make their way into everyday practice [5]–[8].

Clinical Practice Guidelines (CPGs) are defined as systematically developed statements to assist practitioners and patient decisions about appropriate health care for specific circumstances [9]. CPGs are a major tool for improving the quality of healthcare [10]. To guarantee that CPGs can be an effective tool in healthcare to improve outcome for patients, internationally recognized standards should be developed to assess the quality of CPGs and to promote the rigorous development of CPGs. These standards should be valid, reliable and feasible [11].

The Appraisal of Guidelines for Research and Evaluation (AGREE) instrument was initially developed in 2003, and updated to AGREE II in 2010. Consisting of 6 domains covering 23 key items [12], it is an appraisal tool and validated instrument that has been endorsed by leading producers, raters and compilers of international CPGs to provide a framework for assessing their quality [13]. Previous studies have demonstrated that the quality of CPGs in different clinical areas was modest or variable [13]–[16]. The current study aims to assess the quality of CPGs for MG by the AGREE II instrument, and to stratify the quality according to type of CPGs, performers, and pubtime.

## Methods

### Inclusion and exclusion criteria

We included CPGs that were concerned with all areas of MG and that were published in both journals and the internet, including comprehensive CPGs and others which only concentrated on the management of MG. The following studies were excluded: Chinese versions of foreign CPGs and consensuses and adapted versions of CPGs from other countries; Duplication; Explanation, guidance, evaluation on literature of CPGs, and abstract.

### Literature search

Two foreign websites related to CPGs, as well as English and Chinese major academic databases were systematically searched on September 20th 2013: DynaMed (http://dynamed.ebscohost.com/), the National Guideline Clearinghouse (NGC) (http://www.guideline.gov), Chinese Biomedical Literature database (CBM), PubMed and EMBASE.

### Search terms

myasthenia gravis, guideline*, consensus, standard, criterion.

### Search strategy for PubMed

#1 "myasthenia gravis"[MeSH Terms]

#2 "myasthenia gravis" OR MG[All Fields]

#3 #1 OR #2

#4 guideline* OR consensus OR standard OR criterion[Title/Abstract]

#5 "Guidelines as Topic"[Mesh] OR "Practice Guidelines as Topic"[Mesh] OR "Guideline" [Publication Type] OR "Practice Guideline" [Publication Type]

#6 #4 OR #5

#7 #3 AND #6

### Data extraction and quality assessment

We established a standard table on Microsoft Excel 2003 (Microsoft Corp, Redmond, WA, www.microsoft.com). In addition to the items of AGREE??, the following data were also extracted for each study: title of guidelines, year of publication, organizations or countries of publication, number of authors, number of organizations, updated/period, developed methods, number of references, topics covered and number of guideline pages.

The AGREE?? instrument was used to assess the methodological quality of included CPGs. 23 key items of 6 domains were scored on a scale of 1–7, with 1 being strongly disagree and 7 being strongly agree. The score for each domain is obtained by summing all the scores of the individual items in a domain and then standardizing as follows: (obtained score - minimal possible score)/(maximal possible score - minimal possible score). A guideline is “strongly recommended” if the majority of items (above 4 items) scores are above 50%. A guideline is “recommended” if 3 main items scores are above 50%. A guideline is “weakly recommended” if 1–2 items score above 50%. A guideline is “not recommended” if all items score below 50%.

The data extraction and quality assessment were performed independently by two trained reviewers. Disagreement between reviewers was resolved through consensus or by consulting an independent expert adjudicator.

### Statistical analysis

A descriptive statistic analysis was used for the total score by each reviewer and score per domain. In order to assess inter-rater reliability within each domain, the value of intraclass correlation coefficients (ICCs) was calculated [17]. Statistical significance was set at P<0.05 and the software used for analysis was SPSS 17.0.

In order to assess the quality according to type of CPGs, the date of publication, and performers, quality scores of evidence-based (EB) and non-evidence-based (non-EB) CPGs, scores of CPGs published before and after AGREE II instrument and scores of CPGs performed by AAN, NGC or other organizations were compared by a t-test.

## Results

### Guidelines characteristics

**Figure 1:**
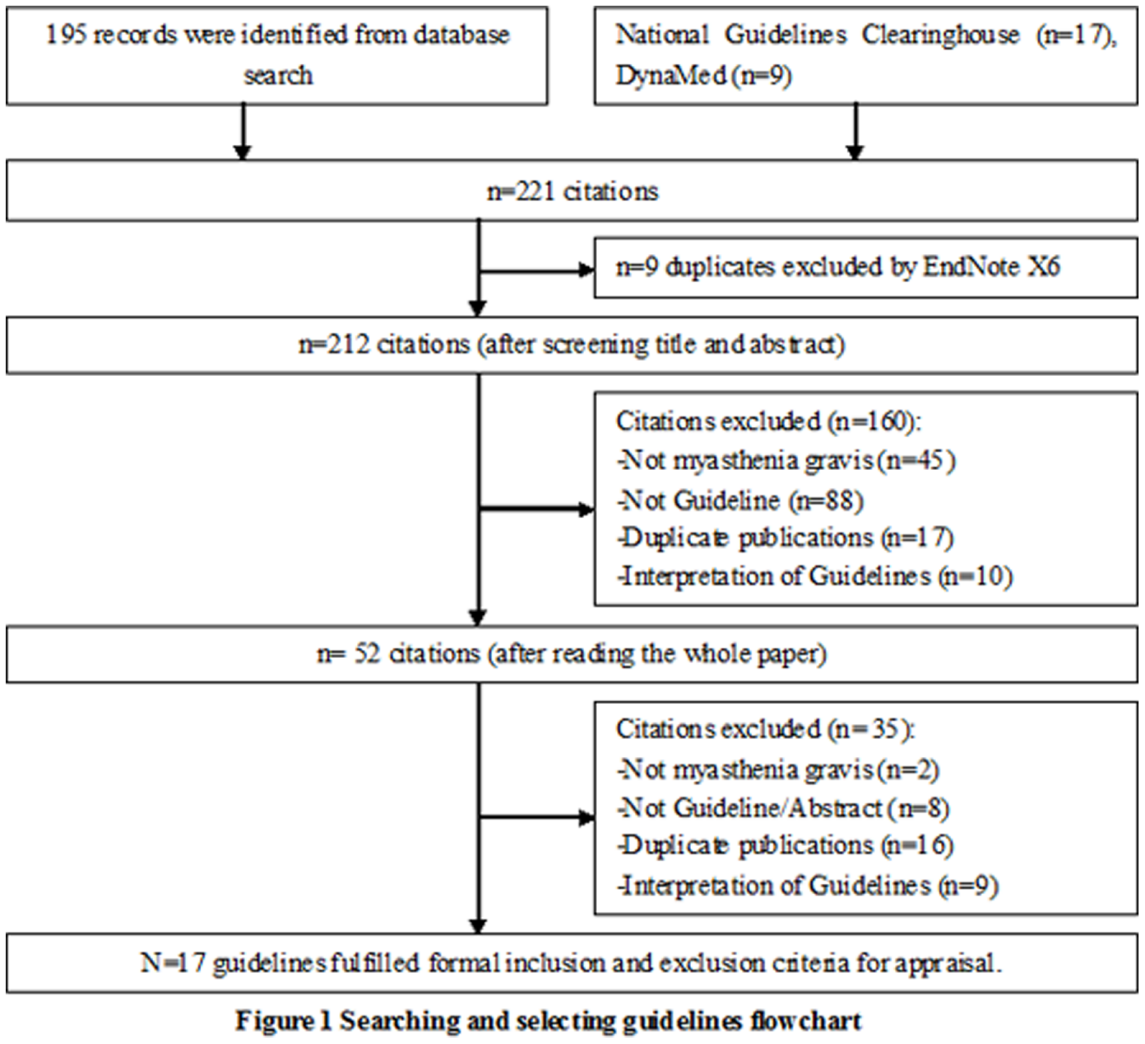
Searching and selecting guidelines flowchart.

A total of 221 records were identified and screened through a computerized search and website consultation performed with the agreed search terms. 17 CPGs [18]–[34] that met the inclusion criteria were identified (details for literature selection can be seen in Figure 1).

The demographic characteristics for each of the included guidelines are presented in Table 1. The data extraction was performed by two reviewers, with 3 CPGs discussed due to the disagreement of topics covered and development methods. All guidelines were developed between 1999 and 2012, with 52.94% of guidelines developed from 2009 onwards. Most of the CPGs (58.82%) were developed by US-based organizations and 2 CPGs (11.76%) were developed in China. 11 CPGs (64.71%) were evidence-based guidelines. The majority of CPGs (88.24%) reported the number of authors and 8 CPGs (47.06%) had more than 10 authors. 4 CPGs (23.53%) recorded the details of any CPG guideline changes and updates. The average total number of pages of the CPGs was 10.47 (range: 2–51).

**Table 1:** The basic characteristics for included 17 CPGs.

Authors/Titles	Year	Country	Number of authors	Number of affiliations	Topics covered	Updated/period	Number of references	Number of pages	Developed methods
Marinos CD [18]	1999	American	1	2	Treatment	Not mentioned	154	19	Evidence-based
Donofrio PD [19]	2009	American	8	Unclear	Treatment	Not mentioned	112	11	Evidence-based
Benatar M [20]	2012	American	9	9	Treatment	1 times/12 years	50	9	Evidence-based
Adekanye O [21]	2009	European	Unclear	Unclear	Treatment	Not mentioned	5	2	Literature review
Benatar M [22]	2007	American	2	Unclear	Treatment	Not mentioned	20	6	Evidence-based
Consensus of diagnosis and treatment for myasthemia gravis [23]	2011	China	Unclear	2	Treatment, diagnosis	Not mentioned	22	5	Literature review
Consensus of diagnosis and treatment for myasthemia gravis [24]	2012	China	43	2	Treatment, diagnosis	Not mentioned	18	8	Literature review
Gronseth GS [25]	2000	American	15	Unclear	Treatment	3 times/10 years	44	6	Evidence-based
Elovaara I [26]	2008	American	9	14	Treatment	Not mentioned	15	7	Evidence-based
Skeie GO [27]	2010	American	10	14	Treatment	1 times/4 years	94	9	Evidence-based
Cortese I [28]	2011	American	16	Unclear	Treatment	1 times/15 years	28	9	Evidence-based
Patwa HS [29]	2012	American	14	Unclear	Treatment	Not mentioned	36	9	Evidence-based
AAEM [30]	2001	USA	33	NR	Diagnose	Not mentioned	NR	3	Literature review
Feasby T [31]	2007	Canadian	11	11	Management	Not mentioned	129	51	Evidence-based
Skeie GO [32]	2006	Europe	10	10	Treatment	Not mentioned	78	9	Evidence-based
Willison HJ [33]	2000	Europe	7	7	Diagnose	Not mentioned	25	4	Literature review
Bascić-Kes V [34]	2012	Europe	7	2	Management	Not mentioned	81	11	Literature review

### Overall quality Assessment of guidelines

6 CPGs [25]–[29], [31] were strongly recommended as a result of the majority of item (above 4 items) scores being above 50%. These CPGs were produced mainly by the National Guideline Clearinghouse (NGC). One guideline [22] is recommended due to 3 main item scores being above 50%. One CPG [24] was not recommended due to all item scores being below 50% (see Table 2).

**Table 2:** The results of quality assessment for each guideline.

Authors/Titles	the Score of Each Item (%)						Overall Recommendation
	Scope&Purpose	Stakeholders	Rigor	Clarity	Applicability	Editorial	
Marinos CD [18]	52.78	22.22	13.54	52.78	20.83	0.00	weakly recommended
Donofrio PD [19]	61.11	38.89	31.25	55.56	27.08	20.83	weakly recommended
Benatar M [20]	66.67	33.33	18.75	61.11	10.42	29.17	weakly recommended
Adekanye O [21]	66.67	36.11	4.17	50.00	8.33	8.33	weakly recommended
Benatar M [22]	69.44	52.78	32.29	63.89	12.50	41.67	recommended
Consensus of diagnosis and treatment for myasthemia gravis [23]	33.33	19.44	14.58	52.78	12.50	0.00	weakly recommended
Consensus of diagnosis and treatment for myasthemia gravis [24]	27.78	22.22	16.67	41.67	10.42	0.00	not recommended
Gronseth GS [25]	75.00	50.00	83.33	77.78	52.08	45.83	strongly recommended
Elovaara I [26]	77.78	61.11	68.75	77.78	60.42	66.67	strongly recommended
Skeie GO [27]	80.56	72.22	77.08	83.33	68.75	41.67	strongly recommended
Cortese I [28]	72.22	69.44	83.33	83.33	47.92	75.00	strongly recommended
Patwa HS [29]	72.22	66.67	75.00	88.89	43.75	75.00	strongly recommended
AAEM [30]	52.78	13.89	21.88	47.22	14.58	4.17	weakly recommended
Feasby T [31]	77.78	77.78	66.67	77.78	54.17	58.33	strongly recommended
Skeie GO [32]	55.56	44.44	43.75	69.44	37.50	33.33	weakly recommended
Willison HJ [33]	61.11	47.22	29.17	41.67	25.00	4.17	weakly recommended
Bascić-Kes V [34]	47.22	38.89	30.21	58.33	22.92	4.17	weakly recommended

### Scope and Purpose

This domain includes the overall objectives of the guidelines, the health questions covered by the guidelines, and the population for whom the guidelines are intended [12]. The range and mean ± SD of the overall quality score for this domain were 27.78%–80.56% and 61.76% ±15.29% respectively. Only 3 CPGs scored below 50% for this domain. 82.35% of the criteria of this domain were satisfied, although Chinese CPGs had poor reporting for this domain. The ICCs showed moderate agreement between reviewers (ICC  = 0.738, 95% Confidence Interval [CI]  = 0.277–0.905).

### Stakeholder involvement

This domain assesses whether or not the compositions of the working group were represented, the patients' views and preferences on the development of guideline have been sought, and whether target users and pretesting among end users have been correctly defined [12]. The overall score in this domain was poor with a mean of 45.1% ±19.65% (range: 13.89%–77.78%). 10 out of 17 CPGs (58.82%) scored below 50%. The ICCs showed moderate agreement between reviewers (ICC  = 0.802, 95%CI: 0.454–0.928).

### Rigor of development

This domain is the core of the guidelines methodology, involving eight items. It relates to “the process for synthesizing and gathering the evidence, and the methods used for formulating the recommendations and to update them”. The mean score ± SD for this domain was 41.79% ±27.56%, and the range of overall quality score was 4.17%–83.33%. Only 35.3% of guidelines scored above 50%. The ICCs were high (ICC  = 0.959, 95%CI: 0.888–0.985).

### Clarity of presentation

This domain focuses on whether or not recommendations are specific and unambiguous, different options for management of the condition or health issue are clearly presented, and key recommendations are easily identifiable. 82.35% of CPGs scored above 50%. The range and mean ± SD of the overall quality score for included CPGs were 41.67%–88.89% and 63.73% ±15.42%, respectively. The ICCs were 0.704 (95% CI: 0.181–0.953).

### Applicability

This domain is concerned with guideline implementations which include organizational barriers, cost implications and monitoring criteria [10]. Only 4 of 17 CPGs (23.53%) scored above 50%. The range and mean ± SD of overall quality score for included CPGs were 8.33%–68.75% and 31.13% ±19.84% respectively. The ICCs were high agreement (ICC  = 0.871, 95% CI: 0.644–0.958).

### Editorial independence

This domain evaluates how funding bodies influence the guideline content and whether the interests of all CPG contributing members have been recorded and addressed. The assessment result shows that 23.53% of CPGs scored above 50%. The range and mean ± SD of overall quality score for included CPGs were 0–66.67% and 29.90% ±27.37% respectively. The ICCs showed high agreement between reviewers (ICC  = 0.884, 95% CI  = 0.681–0.958).

### Statistical analysis according to type of CPGs, performers, and pubtime

Of the 17 CPGs assessed, 11 were EB CPGs. The other 6 were considered non-EB CPGs. Table 3 showed that EB CPGs had higher quality scores for all of the AGREE domains when compared with non-EB CPGs, and the difference was statistically significant for all domains (P<0.05). All domain scores appear slightly higher for CPGs published after the development and validation of the AGREE II instrument (2010). However, the difference results are statistically insignificant for all domains (P>0.05) (Table 3).

**Table 3:** Comparison of mean quality score for each AGREE II domain by subgroup.

Subgroups	Domain(Mean ± SD)					
	Scope&Purpose	Stakeholders	Rigor	Clarity	Applicability	Editorial
Year of publications						
Pre-AGREE II(n = 10)	65.00±9.82	44.44±18.33	39.48±25.80	61.34±13.76	31.24±18.76	28.33±24,27
Post-AGREE II(n = 7)	57.14±20.90	46.03±22.90	45.09±31.71	67.06±18.10	30.95±22.84	32.14±33.22
p values	0.35	0.88	0.7	0.48	0.98	0.8
MD,95%CI	7.86 (−8.78, 24.49)	−1.59(−22.00,18.83)	−5.61(−34.03,22.81)	−5.67(−21.57,10.22)	0.30(−20.23,20.83)	−3.81(−32.66,25.04)
Performers						
NGC&AAN(n = 7)	72.62±6.29	58.73±12.04	64.43±22.87	75.79±11.86	44.64±19.31	52.38±20.39
Others(n = 10)	54.17±15.28	35.56±18.56	25.93±18.00	55.28±11.67	21.67±14.47	14.17±19.66
p values	0.0006	0.002	0.0002	0.0004	0.008	0.0001
MD,95%CI	18.45(7.89,29.01)	23.17(8.62,37.73)	38.50(18.21,58.78)	20.52(9.13,31.90)	22.98(6.09,39.86)	38.21(18.81,57.62)
EB CPGs						
Yes(n = 11)	69.19±9.25	53.54±17.66	53.98±26.54	71.97±12.14	39.58±19.59	44.32±23.37
No(n = 6)	48.15±15.28	29.63±12.99	19.44±9.81	48.61±6.51	15.63±6.81	3.47±3.14
p values	0.002	0.001	0.0001	<0.00001	0.0002	<0.00001
MD,95%CI	21.04 [7.65, 34.44]	23.91(9.18,38.63)	34.53(16.99,52.07)	23.36(14.49,32.23)	23.96(11.16,36.75)	40.85(26.81,54.88)

Notes: MD, mean difference; CI, Confidence interval; EB, evidence-based; CPGs, clinical practice guidelines; SD, standard deviation.

7 of 17 CPGs were developed by the National Guideline Clearinghouse (NGC) or American Academy of Neurology (AAN), and the other 10 CPGs were developed by the National Institute of Neurological Disorders and Stroke, National Institutes of Health of USA (1 CPG), Myasthenia Gravis Foundation of America (1 CPG), The Association of Anesthetists of Great Britain and Ireland (1 CPG), The American Association of Electrodiagnostic Medicine (1 CPG), The IVIG Hematology and Neurology Expert Panels (1 CPG), AD Hoc Committee of the Croatian Scociety for Neurovascular Disorders (1 CPG), European Federation of Neurological Societies (EFNS, 2 CPG), the Neural Immune Group of Neurology Branch of Chinese Medical Association and the Nerve Immunology Branch of Chinese Immunology Association (2 CPGs). The quality scores of CPGs were developed by NGC/AAN and were higher than CPGs developed by other organizations for all domains. The difference was statistically significant for all domains (P<0.05) (Table 3).

### Discussion

According to our data, the overall quality of CPGs for MG is acceptable for two AGREE II domains: scope and purpose, and clarity of presentation (mean score above 50%). The remaining four domain scores are between 27.78% and 40.93%. 6 of 17 CPGs were strongly recommended. Compared with non-EB CPGs of MG, the EB CPGs had statistically significant higher quality scores for all of the AGREE domains. There were no differences for the quality between pre-AGREE II and the post-AGREE II instrument. This would suggest that for some reason the uptake of AGREE-II has not been that good in the MG community. We found that the best performers were CPGs published and endorsed by the AAN, or registered in the NGC. As such, it is recommended that the editors should use the AGREE-II as a compulsory checklist for publishing guidelines to improve on this.

Compared with the world average [35], the mean scores of CPGs for MG were higher for some domains (stakeholder involvement 45.10% vs. 35.0%, clarity of presentation 63.73% vs. 60.0%, and applicability 31.12% vs. 22.0%). The other domains had a slightly lower score (scope and purpose 61.76% vs. 64.0%, rigor of development 41.79% vs. 43.0%, and editorial independence 29.90% vs. 30.0%). We hypothesize that these inconsistencies could be related to the inclusion of Chinese CPGs and non-EB CPGs in our study.

Our study found that the domain with the lowest score was editorial independence (29.90%). Only 2 CPGs reported the detailed information on potential conflicts of interest, with most CPGs (88.24%) reporting as the information as “not stated” or “the authors report no conflicts of interest”. This may be avoided by making it mandatory for authors to provide information that addresses the detailed process listed in the documents. It is noteworthy that the mean score for this domain was “0” regarding the two Chinese CPGs that were included.

The results of the AGREE-II assessment also showed the substandard methodological quality of CPGs in terms of “applicability”. The main flaws presented were lack of advice and/or tools on how the recommendations could be put into practice (64.70% of CPGs did not have any information for this item), and potential resource implications and barriers to their application (70.59% of CPGs did not have any information for this item). It has been widely shown in other diseases that it is hard for clinicians to change their practice after guidelines become available. As such, it is suggested that a pilot test for the applicability of new guidelines should be performed before publication to ensure their feasibility in clinical practice. Moreover, the journal should ask the guideline developer to provide the results of any such pilot test before accepting for publication. 2 of the CPGs [26], [31] were strongly recommended (scoring above 50%) as an example to develop future CPGs for this domain in our study.

The rigor of CPG development is considered to be the most important domains in the assessment of all guidelines, with a list of key items focusing on the methodology employed by the developers, starting from the literature search through to the updating procedure. The mean score for this domain was low. The quality may be improved by involving search experts and methodologists in the guideline development process, as well as clarifying the methods of guideline development [36]. The guidelines should be updated every 3 years [37]. In our survey, only 4 guidelines described updating procedures, and the stated updating periods were 1 time/12 years [20], 3 times/10 years [25], 1 time/4 years [27], and 1 time/15 years [28].

The mean score for stakeholder involvement was 45.1%. Most CPGs did not report any information related to the views and preferences of the target population and target users of the guidelines remained generally undefined. For this, an explicit mechanism for involving the target population should be conducted before guideline development. For example, the views of patients involved in guideline development can be achieved through actively contacting patient groups and patient representatives [36].

The mean quality scores of scope and purpose (61.76%) and clarity of presentation (63.73%) were better than all other domains for this study. Most CPGs described the overall objective and their specific and focused clinical questions. The accurate reporting of the target population was flawed due to incomplete reporting information on data such as age range, clinical details and gender. The overall quality scores of scope and purpose were above 50% for almost all foreign CPGs. Unfortunately, the overall quality scores of the Chinese CPGs were low at 33.33% [23] and 27.78% [24] respectively.

The most significant difference between CPGs is the process by which developers used the same evidence to produce different guidelines [38]. We assessed the CPGs related to MG by applying the AGREE II instrument, and found that the scores of CPGs developed by the AAN or registered in the NGC were statistically higher than other organizations or individual. A variety of studies concluded that there were better scores for EB CPGs when compared with non-EB CPGs [10], [13]. Our study showed that the quality of EB CPGs was statistically significantly higher than non-EB CPGs for all domains. The purpose of the AGREE II instrument is to provide a framework for assessing the quality of CPGs and assist CPGs developers to improve the quality and applicability of CPGs. Table 3 showed that the quality of CPGs for MG improved after publication of the AGREE II instrument, the difference was no statistically significant.

Our study has several strengths. Firstly, the latest instrument for guidelines assessment (AGREE II) was used to assess the methodological quality of CPGs related to MG. Secondly, we performed a subgroup analysis and found the potential elements influenced CPG quality. Thirdly, we performed a systematic and complete literature search, included prominent academic databases (PubMed and EMBASE), two websites specifically related to CPGs (NGC and DynaMed) and one Chinese database (CBM). Furthermore, agreement between reviewers was strong (above 70%), ensuring our conclusions were valid and reliable. The review also had its limitations as we only included English and Chinese CPGs, giving no consideration to reports published in other languages. Also, the review only assessed the reporting of the different items and not the content validity of the recommendations.

Overall, we found the quality of CPGs on MG to be acceptable but flawed. The developers of CPGs need to pay more attention to editorial independence, applicability, rigor of development and stakeholder involvement during the development process. The AGREE II instrument should be adopted by guideline developers, especially so in regards to Chinese guideline development.
